# Regulation of the *Flt3* Gene in Haematopoietic Stem and Early Progenitor Cells

**DOI:** 10.1371/journal.pone.0138257

**Published:** 2015-09-18

**Authors:** Giacomo Volpe, Mary Clarke, Paloma Garcìa, David Scott Walton, Alexandros Vegiopoulos, Walter Del Pozzo, Laura Patricia O’Neill, Jonathan Frampton, Stéphanie Dumon

**Affiliations:** 1 Institute of Biomedical Research, College of Medical and Dental Sciences, University of Birmingham, Birmingham, B15 2TT, United Kingdom; 2 DKFZ Junior Group Metabolism and Stem Cell Plasticity, Division of Molecular Metabolic Control (A170), German Cancer Research Center (DKFZ), Heidelberg, Germany; 3 School of Physics and Astronomy, University of Birmingham, Birmingham, B15 2TT, United Kingdom; Emory University, UNITED STATES

## Abstract

The MYB transcription factor plays critical roles in normal and malignant haematopoiesis. We previously showed that MYB was a direct activator of FLT3 expression within the context of acute myeloid leukaemia. During normal haematopoiesis, increasing levels of FLT3 expression determine a strict hierarchy within the haematopoietic stem and early progenitor compartment, which associates with lymphoid and myeloid commitment potential. We use the conditional deletion of the *Myb* gene to investigate the influence of MYB in *Flt3* transcriptional regulation within the haematopoietic stem cell (HSC) hierarchy. In accordance with previous report, in vivo deletion of *Myb* resulted in rapid biased differentiation of HSC with concomitant loss of proliferation capacity. We find that loss of MYB activity also coincided with decreased FLT3 expression. At the chromatin level, the *Flt3* promoter is primed in immature HSC, but occupancy of further intronic elements determines expression. Binding to these locations, MYB and C/EBPα need functional cooperation to activate transcription of the locus. This cooperation is cell context dependent and indicates that MYB and C/EBPα activities are inter-dependent in controlling *Flt3* expression to influence lineage commitment of multipotential progenitors.

## Introduction

The HSC pool is phenotypically defined as KSL (KIT^+^ SCA-1^+^ LIN^-^) cells. This general classification regroups cells that differ with respect to their capacity to reconstitute the haematopoietic system in lethally irradiated mice. Continuing efforts to discriminate long- and short-term HSC (LT-HSC, ST-HSC), multipotential progenitors (MPP) and lymphoid-primed multipotential progenitors (LMPP) have identified different antibody-based strategies relying on the detection or absence of detection of several surface markers. One such strategy uses of a combination of the SLAM markers CD150, CD244, together with CD48 [1] and CD229 [2], another utilises the differential expression or the receptors THY-1.1, VCAM-1 and CD62L within the KSL population [3,4]. The combination of CD34 and FLT3 are used to segregate mouse LT-HSC (KSL, CD34^-^, FLT3^-^) from ST-HSC (KSL, CD34^+^, FLT3^-^) and MPP (KSL, CD34^+^ FLT3^+^). In addition, the expression level of the FLT3 tyrosine kinase receptor can further separate functional subpopulations of KSL cells [5]. In effect, increasing expression of FLT3, first transcriptionally initiated in fully multi-potential HSC [6] distinguishes HSC, MPP and LMPP [3,7]. This expression gradient associates with a functional role for the receptor, which contributes to the cell fate of multipotential progenitors. The role of FLT3 signalling in lineage commitment has been extensively studied since targeted disruption of the *Flt3* locus [8] and bone marrow transplantation assays revealed a reduced ability of stem cells lacking FLT3 to contribute to both B cells and myeloid cells [9]. In line with these observations, FLT3^hi^ LMPP give rise to lymphocytes, granulocytes and macrophages but lack erythro-megakaryocytic potential [10,11]. The studies using a knock out model for the FLT3 Ligand gene (*Fl*) proved most informative regarding the timing and function of FLT3 signalling in early lymphoid development. Contrasting with normal levels of common myeloid progenitors (CMP) and granulocyte-monocyte progenitors (GMP), the severely reduced number of common lymphoid progenitors (CLP) in the *Fl* ^*-/-*^ animals led Sitnicka and colleagues to conclude that a principal function of FLT3 signalling in steady-state haematopoiesis is to promote lymphoid commitment from a multipotent progenitor/stem cell population [12]. Moreover, their follow-up study, comparing *Il7r*α^*-/-*^, *Fl* ^*-/-*^ and the double knock out *Il7r*α^*-/-*^ *Fl* ^*-/-*^ mice, elegantly demonstrated a key function for FLT3 in the LMPP population, independently from IL-7Rα signalling [13]. Taking place at the earliest stage of lymphoid development in the bone marrow, this non-redundant role is essential to the establishment of transcriptional lymphoid priming, although subsequent repression of *Flt3* expression by PAX5 is paramount for B-cell development [14]. The signalling pathway is also tightly controlled in myeloid cells where constitutive activation of the FLT3 receptor provides a leukaemogenic signal and constitutes an adverse prognostic marker in acute myeloid leukaemia (AML) [15,16]. In this leukaemic context, we previously reported that MYB and C/EBPα proteins could both regulate FLT3 expression [17]. If this finding is transferable in the HSC context, it raises the possibility that these factors may influence HSC commitment potential through regulating FLT3 expression during normal haematopoiesis.

Extensive studies demonstrated that MYB plays an essential role during normal haematopoiesis. Mice homozygous for a knock out allele of the *Myb* gene die at embryonic day E15 as a result of a failure to develop an adult blood system [18]. Thus, to facilitate further investigation of the role of MYB in haematopoiesis, mouse models have been generated with knock down (KD) [19,20], mutant alleles [21,22], or conditional inactivation of the *Myb* locus [19,23,24]. Together with chimera studies [25], these models have revealed that perturbation of MYB activity affects haematopoietic stem cell (HSC) maintenance and activity [20,21,24] and skews lineage commitment towards abnormal megakaryocytic and myelo-monocytic differentiation [19,20,23,25–33].

Here, we use conditional deletion of the *Myb* gene [19], to clarify its role in *Flt3* regulation at the early stages of haematopoiesis. In line with previous reports, we find that, in two days, loss of MYB expression leads to the exhaustion of HSC, which associates with an enhanced differentiation rate and loss of proliferation potential. Within the KSL compartment, we show that *Myb* depletion results in a reduction of *Flt3* expression, observable prior to HSC depletion. The latter result could be recapitulated in a haematopoietic stem/progenitor cell line, in which it was possible to demonstrate that the regulation of *Flt3* transcription by MYB and C/EBPα required their functional cooperation. This cooperative regulation involves cis-regulatory regions located in the *Flt3* promoter and first intron, the occupancy of which directly correlates with FLT3 expression in primary HSC.

## Materials and Methods

### Mice

The *Myb* ^*F/F*^ and *Myb* ^+/-^ mice are as described previously [18,19]. 8 week old *Myb* ^*F/F*^:*MxCre* and *Myb* ^*+/+*^:*MxCre* littermates were injected with 250μg poly(I:C) (Sigma). The colony was maintained on a C57BL/6 background. Animal experiments were performed under a UK Home Office Project License held by JF, which underwent ethical review through the University of Birmingham ethical review committee. The study involved sacrifice of mice, performed by dislocation of the neck as a Schedule 1 killing procedure according to UK Home Office regulations.

### Flow cytometry antibodies

Single-cell bone marrow suspensions were depleted from red cells by selective lysis. The lineage cocktail including anti-CD5, CD8a, CD11b, B220, Gr1 and Ter119, FITC or APC conjugated was combined with KIT_PE-Cy5 and SCA-1_PE-Cy7. Analysis of KSL subpopulations used FLT3_PE, VCAM_eFlour450 and CD62L_APC (eBioscience). Analysis of LMPP presented in S2 Fig was performed using SCA-1_APC, KIT_PE-Cy5, CD150_PE-Cy7, CD48_eFluor450 and FLT3_PE (eBioscience). Non-specific binding was prevented using anti-CD16/32, aside from progenitor analysis where CD16/32_PE was used together with CD34_FITC and lineage_APC (eBioscience).

### Colony assays

For colony assay, 500 FACS-sorted KSL cells from both *Myb* cKO and WT mice were seeded in methylcellulose M3434 medium (Stem Cell Technologies Inc.). After 6 days, colonies were counted and scored based on their size. For the differentiation assay, sorted KSL cells were cultured for 48h in Iscove’s Modified Dulbecco Medium (IMDM) with 10% horse serum (Sigma-Aldrich), L-glutamine (2 mM), penicillin (50U/ml), murine SCF (20ng/ml), FL (10ng/ml) and TPO (5 ng/ml).

### Nuclease hypersensitive site mapping

Cell nuclei were prepared in 1ml digestion buffer (15mM Tris-HCl pH7.5, 15mM NaCl, 60mM KCl, 5mM MgCl_2_, 300mM glucose, 0.5mM EGTA, 0.1%NP40) and partially digested 10 minutes at 37°C with DNase I (0 to 60units). A stop solution (0330μl of 100mM EDTA / 4% SDS) was added to terminate the reaction. RNA and proteins were sequentially digested at 37°C with 100μg RNase A (1hour) and 100μg proteinase K (overnight). Extracted DNA was used as template for Q-PCR reactions. When using small numbers of cells, all volumes and quantities were reduced by a scale factor of 2.5. Ratios between Q-PCR results from partially digested and untreated samples (2^-ΔCt^) reflect the extent of nuclease sensitivity across regions covered by PCR amplicons.

### X-ChIP

X-ChIP assays were performed as previously described [34], using antibodies from Santa Cruz Biotechnology and anti-MYB antibody from Upstate Ltd (Merck Millipore)

### Quantitative PCR

Q-PCR reactions for *Myb*, *Flt3*, *Dntt*, *Il7r*α, *Notch1* and *Hprt*, *gapdh and β*_*2*_ *-microglobulin* were performed using predesigned Taqman gene expression assays (Applied Biosystems). Primers for nuclease hypersensitivity assays and X-ChIP were previously described [17].

### Transfections

For the HPC7 line, 5 x 10^6^ cells were electroporated with up to 6μg of DNA using the Amaxa 3D-nucleofector with the solution L and program X-001, or the Amaxa 4D-nucleofector with the SF cell line solution and the DS-120 program (Biosystems), respectively. C/EBPα and MYB expressing plasmids or corresponding shRNA vectors (Origene) were co-transfected with a GFP-encoding vector (Amaxa) and GFP-expressing cells were sorted on a MoFlo XDP cell sorter (Beckman Coulter).

Transfections of primary cells were performed on a population enriched in bone marrow KIT^+^ cells. Following the enrichment step using a MoFlo XDP cell sorter (Beckman Coulter), the cells were cultured 2 hours prior to transfection in IMDM medium supplemented with 5%FBS, 50u/ml penicillin, 50μg/ml streptomycin, 0.1mM non-essential amino acids, 2mM L-glutamine, 1mM sodium pyruvate, 50μM 2-mercaptoethanol, 10ng/ml GM-CSF, 25ng/ml SCF, 25ng/ml IL11, 10ng/ml IL3, 25ng/ml TPO and 4u/ml EPO. We used the Amaxa 3D-nucleofector with the solution P3 Primary Cells and program DS-138 to electroporate sets of 1 to 2 x 10^6^ cells with 0.5μg of the Amaxa pmax GFP plasmid and 600nM siRNA in total. For the single knockdowns 300ng of *Myb* (s70212, Ambion—Lifetechnologies) or *Cebpa* siRNA (s63855, Ambion- Lifetechnologies) were combined with 300nM of siRNA control (4390843 Silencer Select Negative Control #1—Lifetechnologies) to maintain comparability with the double knockdown results. Transfected cells were cultured for 20 hours in the aforementioned IMDM-based medium, analysed by flow cytometry and GFP expressing KSL cells were sorted for quantitative PCR analysis.

### Luciferase assays

The reporter constructs HS-A/HS-B/Luc and HS-A/HS-B/luc/HS-C were generated by cloning the *Flt3* regulatory regions at positions -1459bp to +113bp (including HS-A and HS-B) and +7227bp to +8063bp (encompassing HS-C) with respect to the Flt3 ATG, into the pGL3 basic vector (Invitrogen). HPC7 cells were electroporated with 3μg luciferase reporter construct, 2μg expression vector for the MYB or C/EBPα factor or corresponding control vector, and 1μg of β-galactosidase reporter plasmid. Luciferase activities were measured 24h later as previously described [35]. Luciferase readings were normalised against β-galactosidase activity (Galacton kit, Applera). Identical series of transfection were performed for each separate experiment and results were normalised to 100 to allow comparability of the resulting patterns and minimise artificial variability introduced by the use of different batches of reagents.

### Statistical analysis

Statistical significance was determined by performing the Student’s t-test. The tests were paired when relating to transfection experiments on cell lines. All t-tests were 2-tailed but for the luciferase-based transactivation assay, for which the test was 1-tailed with a null hypothesis of no increase in activity. The null hypothesis is rejected when p<0.05 and p values were graded with stars as indicated in the caption of figures.

## Results

### A 24 hour window permits investigation of early events following *Myb* gene deletion

**Fig 1:**
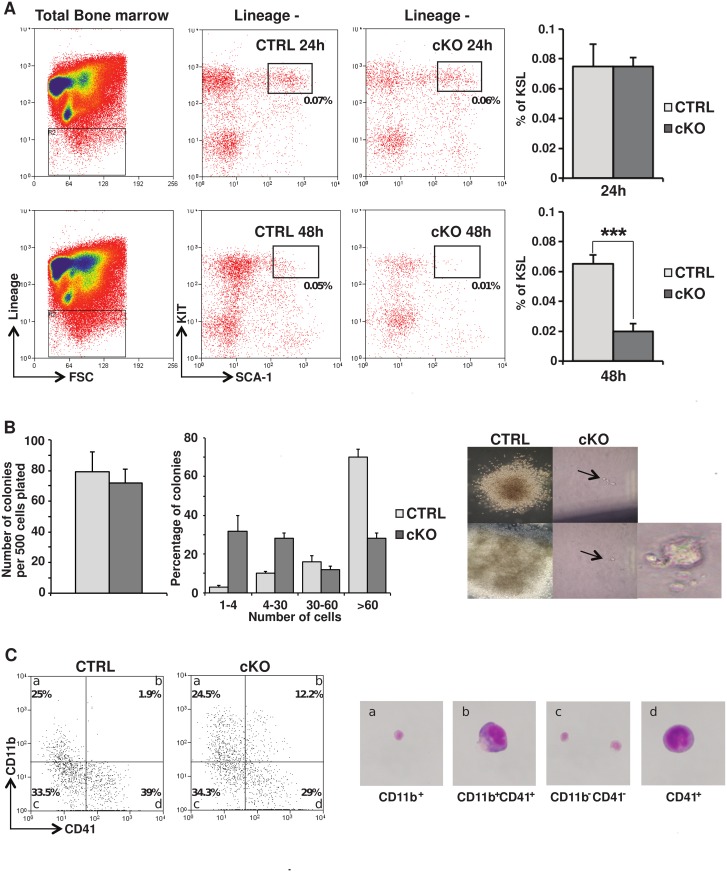
MYB deletion affects KSL cell number and differentiation. (A) Flow cytometric analysis of bone marrow from *Myb* ^*F/F*^:*MxCre* mutant mice and *Myb* ^*+/+*^:*MxCre* control animals, 24 or 48 hours following in vivo induction of the *Myb* ^*F/F*^ allele deletion by p(I:C) injection. The representative two-dimensional plots show the gating on lineage negative cells and the typical KIT^+^/SCA-1^+^/LIN^-^ (KSL) staining. Histograms represent the percentage of KSL cells within the total bone marrow population, with numbers presented as mean ± SEM, determined from 3 independent experiments (***, p<0.0001). (B) Control and *Myb*-deleted KSL were sorted and seeded in methylcellulose. Colony numbers and size were assessed after 6 days. The right histograms represent total colony numbers and the relative proportions of colonies based on size respectively. Pictures of the counted colonies are presented in the left panels. (C) Sorted KSL from control and *Myb*-deleted animals 24 hours post injection of poly(I:C) were cultured into liquid medium containing SCF, FL and TPO. After culturing for 2 days, the cells were re-stained and analysed by flow cytometry for expression of CD11 and CD41 (left panel). Cells from the *Myb* ^*F/F*^:*MxCre* bone marrow were sorted on the basis of CD11 and CD41. The sorted cells were spun onto glass slides and stained with Diff-Quick (upper right panel). The images show representative cells demonstrating monocytic (CD11b^+^) and megakaryocytic (CD11b^+^CD41^+^ and CD11b^-^CD41^+^) morphologies.

A previous report from Lieu and Reddy [24] showed that in vivo *Myb* deletion results in HSC depletion due to impaired proliferation and accelerated differentiation. To test if this system could allow to investigate earlier phenotypes associated with loss of MYB function, we induced conditional deletion of the *Myb* gene in *Myb* ^F/F^ mice expressing CRE recombinase from the *MxCre* transgene by means of poly(I:C) injections. As expected, *Myb* deletion resulted in a significant decrease in bone marrow cellularity at 48 hours in comparison to the *Myb* ^+/+^ control littermates (data not shown) and a severe reduction of the relative proportion of the KSL population (Fig 1A). However, these cellular losses were not seen at 24 hours post poly(I:C) injection. Annexin V staining confirmed that cell death was not increased within the *Myb* deleted progenitors as previously reported [33] and demonstrated that apoptosis was neither changed in KSL or LMPP-enriched compartments both at 24 hours and 48 hours post poly(I:C) injection (S1 Fig). To assess the behaviour of single cells at 24 hours post poly(I:C) injection, both in terms of proliferation and differentiation, we plated sorted KSL cells from cKO and littermate controls in semi solid medium sustaining multilineage haematopoietic differentiation. After 6 days in culture, the overall number of colonies formed by *Myb* cKO KSL cells and their WT counterparts proved similar (Fig 1B). However, the deletion of *Myb* resulted in a dramatic decline in average colony size with a large number of small clusters of 1 to 4 cells generally displaying megakaryocytic-like features (Fig 1B). Accordingly, when placed in liquid cultures, *Myb*-deleted KSL cells underwent rapid differentiation, acquiring within 48 hours both monocytic (CD11b) and megakaryocytic (CD41) characteristics, sometimes concomitantly (Fig 1C), constituting an abnormal population known to be associated with the *Myb* KD and cKO models [20,24,33]. Thus, this model recapitulate the published characteristics of *Myb* deleted cells and offers a window of opportunity (24 hours post injection) to study the early phenotype associated with the loss of MYB activity.

### *Myb* deletion affects *Flt3* expression in bone marrow HSC

**Fig 2:**
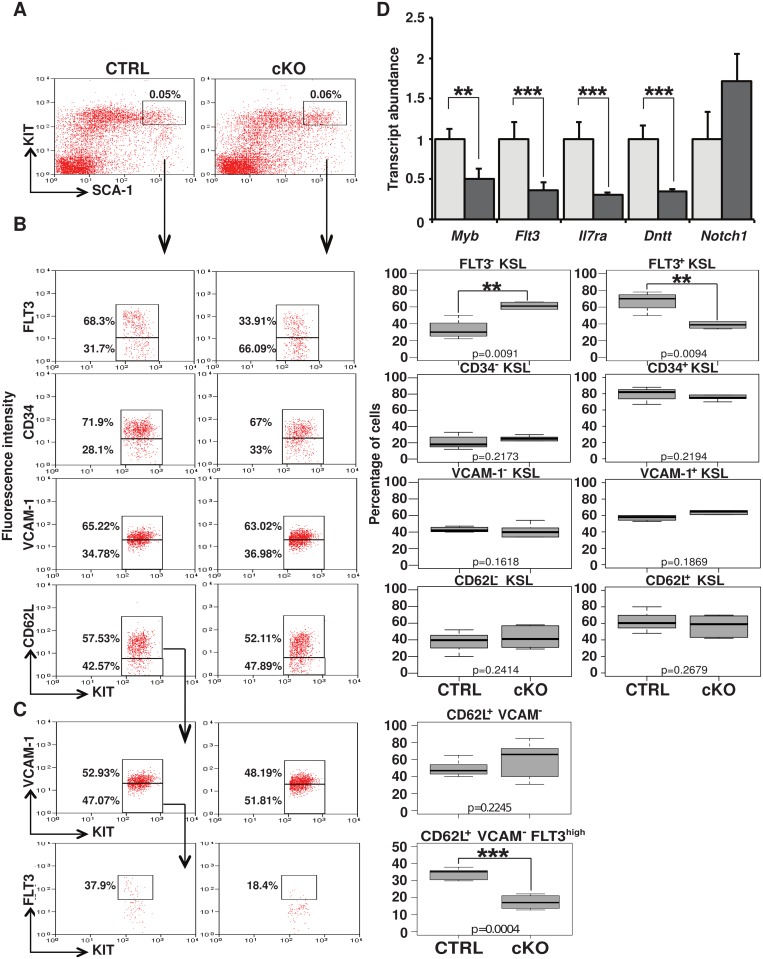
Induced *Myb* ablation results in FLT3 down-regulation in a population highly enriched in LMPP. (A) Representative histogram and two-dimensional flow cytometric plot analysis of cells in the KSL compartment of the bone marrow of the cKO and control mice 24 hours post poly(I:C) injection. (B) The cells were further analysed for surface expression of FIT3 and the proportion of different KSL sub-fractions were assessed based on the surface expression of VCAM-1 and CD62L. Regrouping 7 experiments, the right panel shows their relative contribution in percentage of the gated population, indicated as plot header. (C) Serial gating defined a population highly enriched in LMPP (KSL/CD62L^+^/VCAM^-^) for which the percentage of FIT3^hi^ cells was measured. (D) Quantitative PCR analysis of *Myb*, *Flt3*, *Il7r*α, *Dntt and Notch1* RNA expression in KSL cells isolated from the bone marrow of poly(I:C)-induced *Myb* cKOs and litter-mate controls 24 hours post injection. Expression is normalised to *gapdh* and standardised the *Myb* ^*+/+*^:MxCre control samples, set as 1. Error bars represent the standard error of the mean. Plots are representative of 7 independent experiments. Numbers are plotted as mean ± SEM (***, p<0.0001 and **, p<0.01).

We next analysed FLT3 expression in the KSL subset at 24 hours following conditional gene deletion (Fig 2A). Immunofluorescence staining revealed a rise in FLT3^-^ cells mirrored by a sharp reduction in FLT3^+^ KSL in the *Myb*-deleted mice compared to their littermate controls (Fig 2B upper panel). The observed changes in the FLT3^+^ and FLT3^-^ fractions in the KSL population could either reflect down-regulation of Flt3 expression, or be the indirect consequence of an alteration in the balance between the KSL subpopulations, skewed towards an over-representation of FLT3^-^ immature cells. To distinguish between these possibilities by flow cytometry without the need to use expression of FLT3, we employed the surface receptors CD34, VCAM-1 and CD62L to discriminate the different components of the KSL population (Fig 2B). Our analysis of KSL cells from animals undergoing *Myb* conditional deletion showed no significant variation in the relative representation of the more immature KSL/CD34^-^, KSL/CD62L^-^, or KSL/VCAM^+^ fractions compared to the control siblings (Fig 2B). In addition, we combined staining strategies to further divide the KSL/CD62L^+^ subset based on VCAM-1 expression. Again, the balance between the resulting sub-populations appeared similar in the presence and absence of MYB within the 24-hour window. However, focusing on the KSL/CD62L^+^/VCAM^-^ population, highly enriched in LMPP that are normally FLT3^hi^, we confirmed that *Myb* gene deletion associates with a significant drop in FLT3 level at this time (Fig 2C). Finally, we confirmed this finding using a slam CD150 based strategy (S2 Fig). To examine changes in gene expression, KSL cells were sorted from the bone marrow of the cKO and control animals and used for cDNA preparation. Mirroring the flow cytometry profiles, the Q-PCR analysis showed a decrease in both *Flt3* and *Myb* RNAs. Q-PCR analysis also highlighted a reduction in the lymphoid-associated transcripts *Il7r*α and *Dntt*, as reported by Greig and colleagues [30], but not that of *Notch1* (Fig 2D). Taken together, our results indicate that the observed decrease in FLT3-expressing cells reflects a down-regulation of *Flt3* rather than a change in the cellular distribution within the KSL compartment.

### Occupancy of *Flt3* intronic elements mirrors *Flt3* expression in primary KSL cells

**Fig 3:**
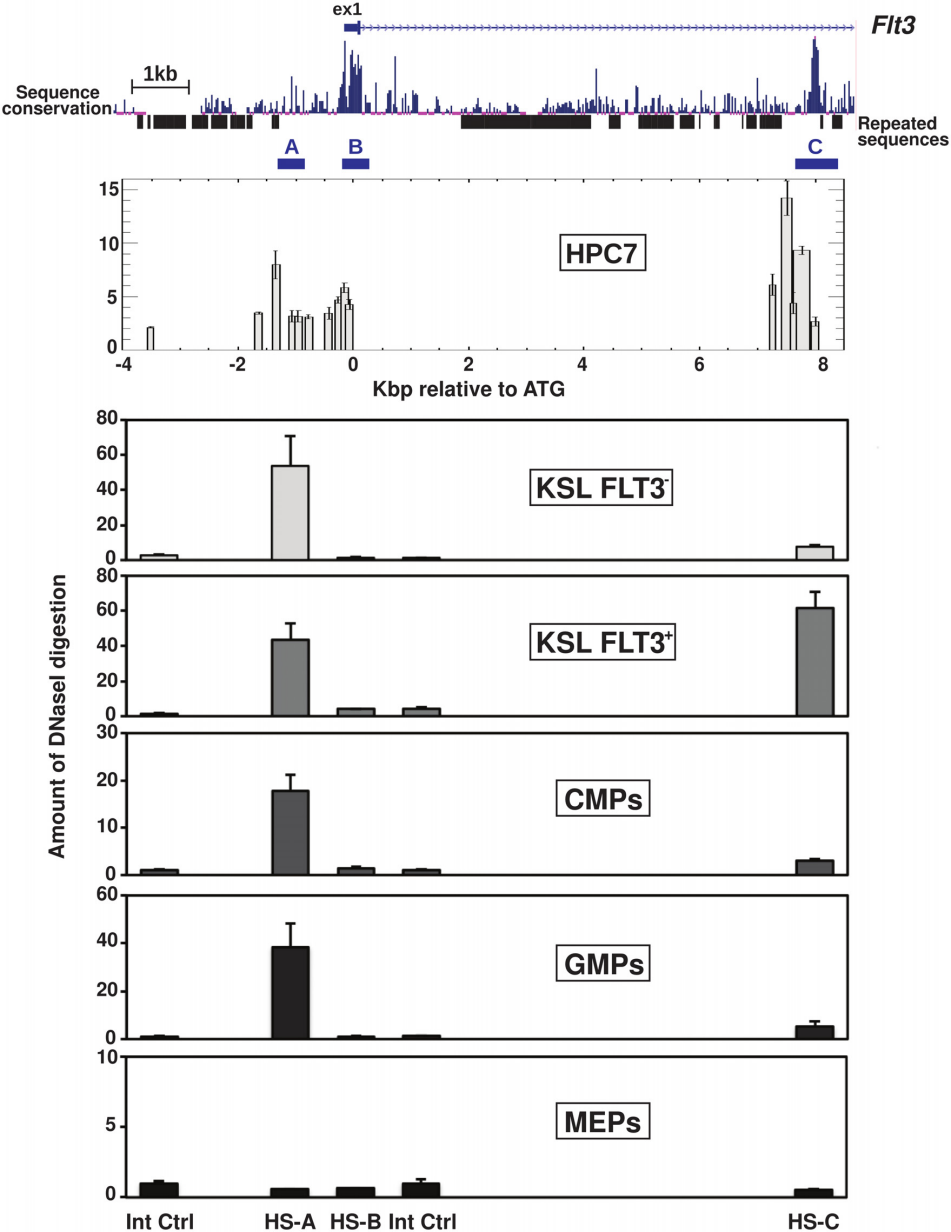
*Flt3* intronic element hypersensitivity coincides with FLT3 expression in primary haematopoietic stem/progenitor cells. Upper panel gives a schematic representation of the murine *Flt3* promoter and first intron (filled box indicates exon1) and shows the level of cross-species sequence conservation and the position of repeated sequences. The underlined regions A B and C indicate the general location of the regions of hypersensitivity to DNAseI digestion in HPC7 cells. Lower panel plots show the assessment of DNaseI digestion sensitivity in the HPC7 cell line and in primary KSL FIT3^+^, KSL FITt3^-^ cells, CMP (LIN^-^/KIT^+^/SCA-1^-^/ CD34^-^/CD16/32^+^), GMP (LIN^-^/KIT^+^/SCA-1^-^/CD34^+^/CD16/32^+^) and MEP (LIN^-^/KIT^+^/SCA-1^-^/CD34^-^/CD16/32^-^). For primary cells, the analysis are restricted to the digestion sites observed in HCP7 at -1.45 kb (HS-A), 0.15kb (HS-B) and +7.5kb (HS-C) from the murine *Flt3* initiation codon.

To investigate *Flt3* regulation in the context of uncommitted early progenitor cells, we applied a similar approach that we used previously in the context of acute myeloid leukaemia [17]. For this purpose, we made use of the multipotent haematopoietic progenitor cell line 7 (HPC7), which was validated as a suitable model system for the analysis of transcriptional programs in early progenitor cells [36,37]. Cell nuclei were treated with increasing quantities of DNaseI and the extracted DNA were analysed by Q-PCR to outline the pattern of digestion. The calculation of the ratio between digested and untreated samples revealed a greater amount of digestion at three cis-regulatory regions; namely HS-A, HS-B and HS-C (Fig 3A). Remarkably, the overall pattern appeared almost identical to that observed in the murine model for acute myeloid leukaemia [17].

To test the relevance of our findings in primary cells, we assayed the nuclease sensitivity of the three identified regions in primary haematopoietic stem/progenitor cells. Nuclei from sorted primary KSL/Flt3^-^ and KSL/Flt3^+^ cells, along with committed CMP (KIT^+^/SCA-1^-^/LIN^-^/CD34^+^/CD16/32^-^), GMP (KIT^+^/SCA-1^-^/LIN^-^/CD34^+^/CD16/32^+^) and megakaryocyte-erythroid progenitors (MEP) (KIT^+^/SCA-1^-^/LIN^-^/CD34^-^/CD16/32^-^) were used for DNaseI assays. The results highlighted a change in nuclease sensitivity at the HS-C site that correlates with the presence of the FLT3 receptor at the surface of the cells (Fig 3). Interestingly, the promoter HS-A region displays hypersensitivity to DNaseI digestion prior to FLT3 protein detection (KSL/FLT3^-^), suggesting that the *Flt3* promoter may be primed for swift activation, mediated through the intronic HS-C region during the transition from ST-HSC to MPP. While this “primed” profile is reinstated in the early stages (CMP and GMP) of the myelo-monocytic lineage, the *Flt3* promoter region appears to be “shut down” upon commitment to the megakaryocyte and erythroid lineages, along which FLT3 expression is never re-established.

### MYB and C/EBPα functionally cooperate to regulate *Flt3* expression in stem/progenitor cells

**Fig 4:**
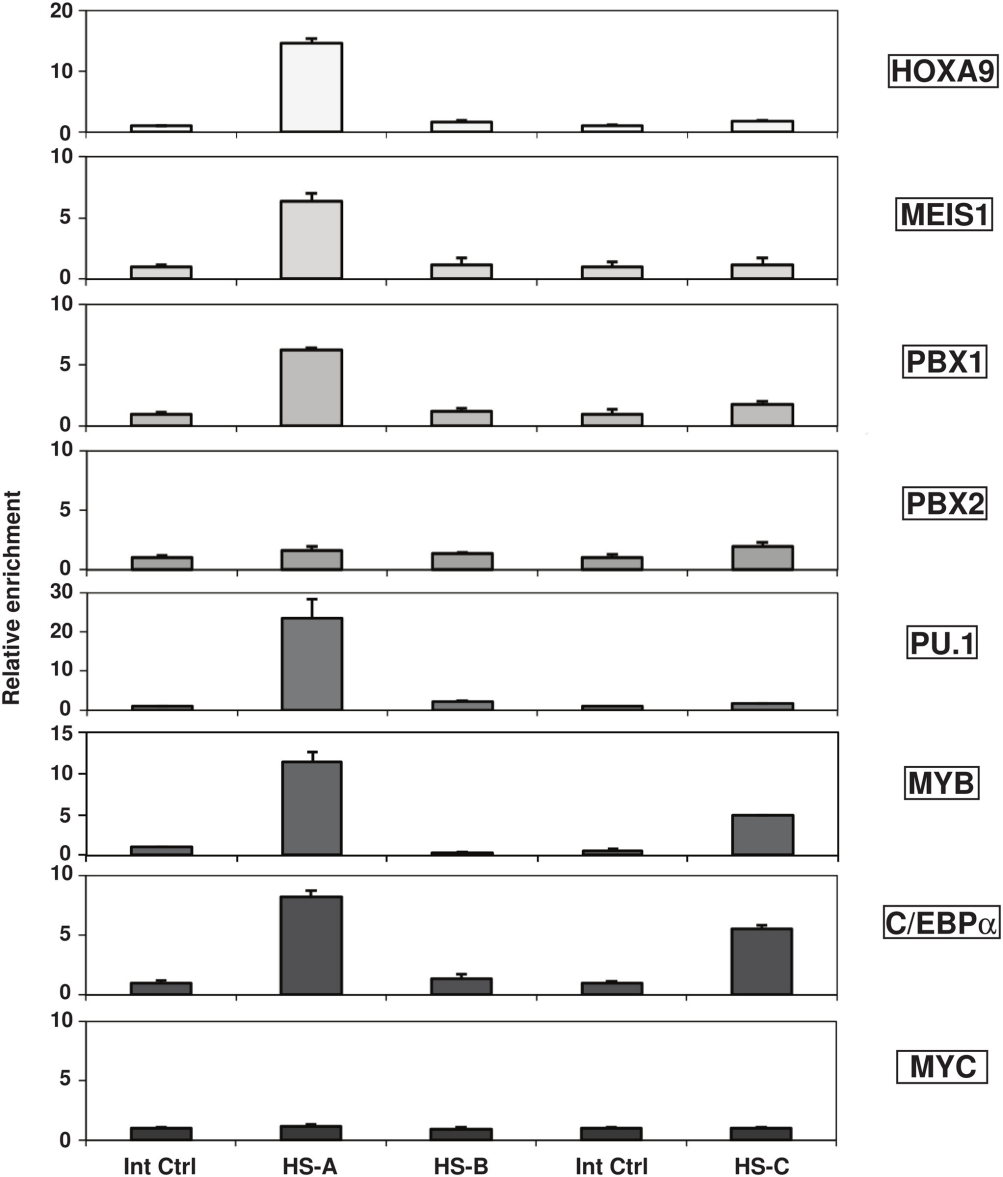
*Flt3* regulatory regions enable the recruitment of a combination of master regulators of haematopoiesis. Detection of in vivo transcription factor binding at the sites of hypersensitivity to nuclease digestion was achieved by ChIP. Relative enrichments from X-ChIP material were determined against the IgG control material by Q-PCR at the location of the hypersensitive regions and normalised against two internal control regions (located at -3.5kb and -0.27kb from the ATG). Error bars represent the standard error of the mean. All plots are representative of a minimum of three independent experiments.

To further investigate *Flt3* regulation in uncommitted cells, we performed a series of X-ChIP experiments in HPC7 and compared the results to our previous findings in myelogeneous leukaemia [17]. The results demonstrated MYB, HOXA9 and MEIS1 binding to the *Flt3* promoter and revealed the co-recruitment of HOX-TALE partner protein PBX1 (but not PBX2) to the HS-A regulatory module in the HSC-like cells (Fig 4). Contrasting with results obtained in the AML context, we find that PU.1 binds promoter HS-A element of the *Flt3* locus in the undifferentiated cells. Furthermore, C/EBPα associates with both HS-A and HS-C and co-localises with MYB in HPC7 cells, whilst these factors are respectively restricted to the intronic element and promoter in murine AML-like cells [17].

**Fig 5:**
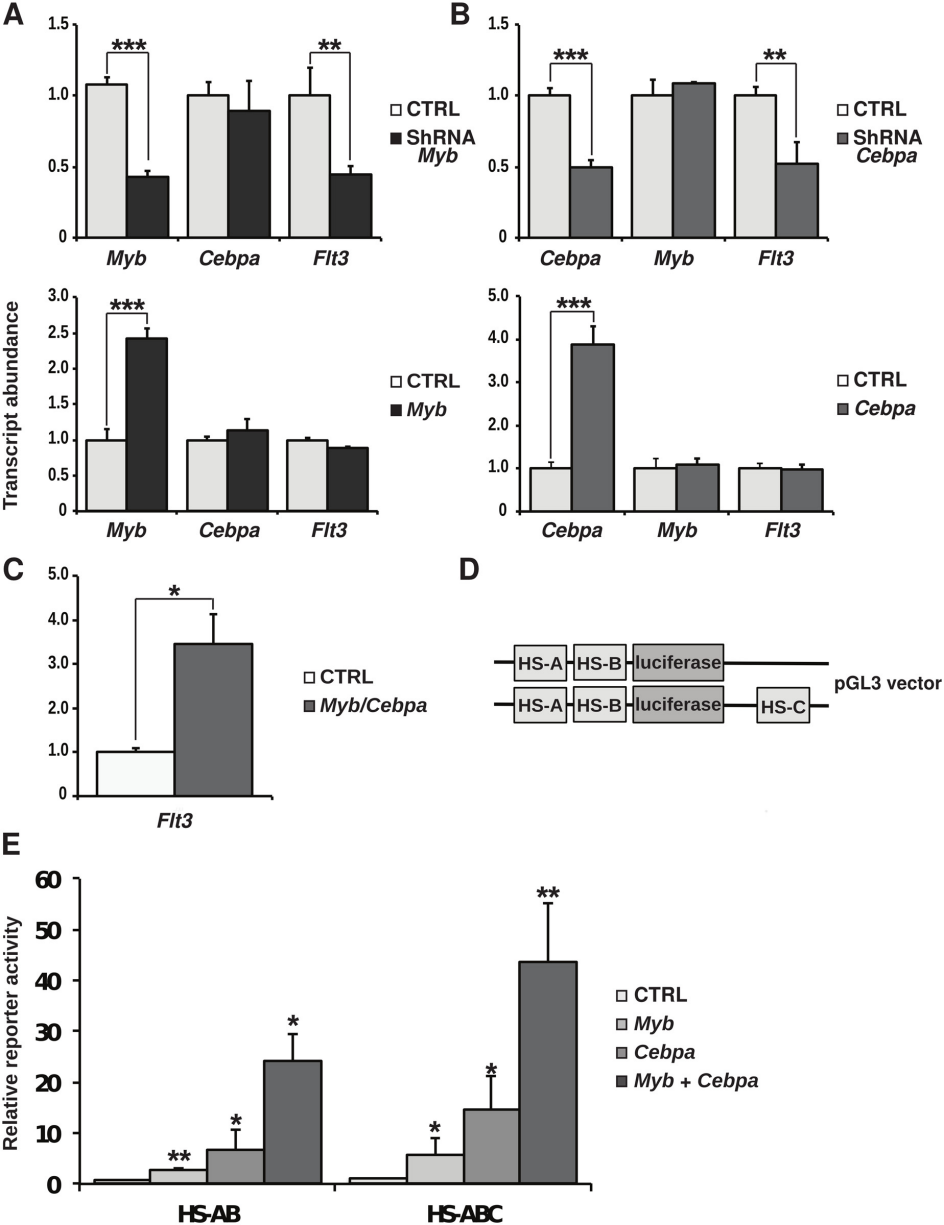
Genetic manipulation and reporter assay studies in HPC7 cells. Quantitative PCR analysis of gene expression in HPC7 cells following shRNA-mediated silencing and ectopic expression of Myb (A) and *Cebpa* (B). (C) Analysis of *Flt3* transcript expression in HPC7 cells co-transfected with *Myb* and *Cebpa* expression vectors or control plasmids. Determined by Q-PCR, *Myb*, *Cebpa* and *Flt3* RNA levels were normalised against *Gapdh* abundance and standardised against the corresponding control transfections. (D) Schematic representation of the reporter constructs in which *Flt3* regulatory regions were placed upstream (HS-A and HS-B) or downstream (HS-C) the luciferase gene. (E) Transactivation assays in HPC7 cells transfected with vector encoding for MYB and C/EBPα proteins. Results were standardised to 100 and bars represent the average proportions across 4 experiments. All results are representative of 3 or 4 independent experiments. P values representations: ***<0.001 and **<0.01.

We next performed shRNA-mediated silencing or enforced expression to evaluate the contribution of MYB and C/EBPα in the regulation of *Flt3* expression in this cellular context (Fig 5A and 5B). Reduction of the expression of either the *Myb* or *Cebpa* genes led to corresponding variations of *Flt3* expression. However, in contrast with our observation in the AML-like cells, over-expression of MYB or C/EBPα alone in the HSC-like cells failed to induce any change in *Flt3* transcript levels. Although, highlighting the need for a functional cooperation between the two factors, enforced co-expression resulted in the up-regulation of the *Flt3* gene (Fig 5C).

We next used luciferase reporter assays to test the relative contribution of the intronic region in the realisation of this cooperative action. The promoter and upstream elements (HS-B and HS-A) were placed upstream of the firefly luciferase reporter. In a second reporter construct, the intronic region (HS-C) was further added downstream of the luciferase gene to mimic its relative position with respect to the transcription start site (Fig 5D). In this artificial setting, both proteins could up-regulate the reporter gene and displayed synergistic activity, which was strengthened in the presence of the *Flt3* intronic enhancer (Fig 5E). Of note, this cooperative activity was not seen in a different cellular context provided by the 293T cells, in which it appeared that the prime effect of C/EBPα was to counter-act MYB activating capacity of the reporter, when co-expressed (S3 Fig).

### Combined knock down of MYB and C/EBPα reduces FLT3 expression in primary stem/progenitor cells

**Fig 6:**
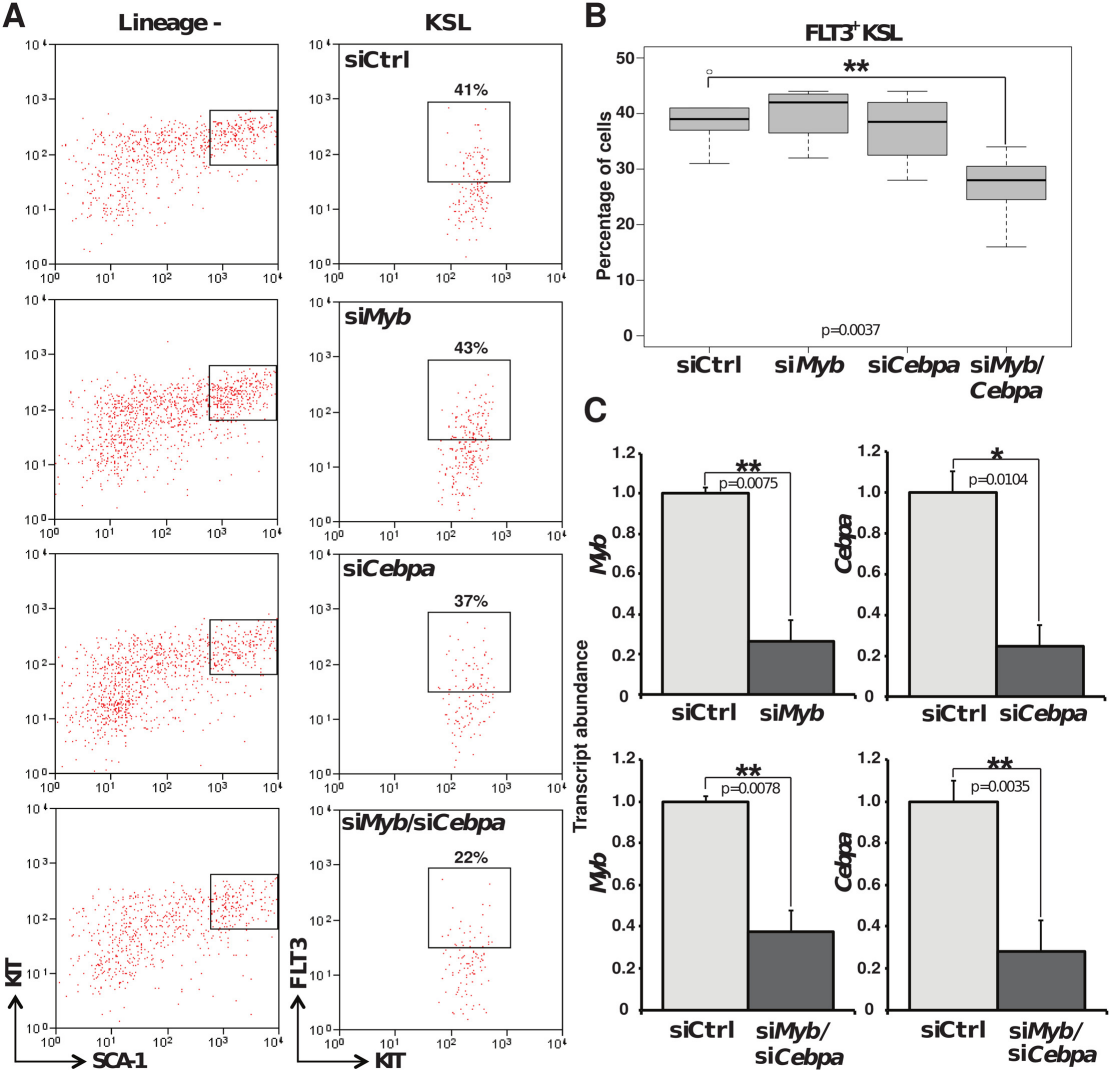
*Myb and Cebpa* knockdown in primary KIT^+^ enriched bone marrow cells. (A) Two-dimensional flow cytometry dot plot showing the analysis of the KSL compartment of a KIT^+^ enriched population transfected with siRNA control or undergoing siRNA-mediated silencing of *Myb* or *Cebpa* for 20 hours. (B) Grouping four independent experiments, boxplots depict the variations in percentage of FLT3^+^ cells within the KSL populations. (C) Quantitative PCR analysis of *Myb* and *Cebpa* RNA expression in sorted transfected KSL cells 20 hours post transfection. Expression is normalised to *β*_*2*_ *-microglobulin* and standardised to the control samples. Error bars represent the standard error of the mean. Plots are representative of 4 independent experiments. Numbers are plotted as mean ± SEM (**, p<0.001 and *, p<0.05).

We investigated the in vivo effect of a reduced level of MYB on FLT3 expression by comparing the percentage of FLT3^+^ KSL cells in *Myb* ^+/-^ mice and wild type littermates. Contrasting with the results obtained in the conditional knock out system, the *Myb* haploinsufficient mice displayed normal amount of FLT3^+^ KSL cells (S4 Fig). We thus hypothesised that some MYB activity may suffice to maintain a normal expression level of FLT3. To test this hypothesis and investigate the effect of reduced C/EBPα level on FLT3 expression in primary stem cells and early progenitors, we proceeded to knock down their expression singly or in combination in a KIT^+^ enriched bone marrow population. Twenty hours after siRNA introduction, the KSL compartment of the transfected cells was analysed to determine the percentage of FLT3^+^ cells. The targeting of *Myb* or *Cebpa* expression alone did not affect the overall expression of the FLT3 receptor (Fig 6A and 6B), despite an efficient reduction of the corresponding transcript levels, which could be verified by quantitative PCR on cDNA from the sorted transfected KSL cells (Fig 6C). However, the proportion of FLT3^+^ was significantly reduced when the expression of both factors was reduced, suggesting that the combinatorial expression of MYB and C/EBPα above a certain level is crucial for FLT3 regulation in primary HSC and early progenitor cells.

## Discussion

We used conditional deletion of the *Myb* gene to analyse MYB influence on FLT3 expression during normal haematopoiesis. As previously described [20,24,33], deletion of the *Myb* gene results in the exhaustion of the HSC pool, observable at 48 hours following poly(I:C) injection. This feature is not seen at 24 hours but we could verify that *Myb* deletion had already occurred as, when cultured, the cells displayed greatly reduced proliferative capacity and accelerated differentiation. At this earlier time, we could observe a reduction of FLT3^+^ cells and showed that this diminution was neither the result of an increase in cell death nor a shift in the balance between the KSL subpopulations. Interestingly, this phenotype only associates with a total loss of expression in the *Myb*-deleted cells, as half dosage or further reduction of *Myb* expression in KSL cells did not result in any apparent change in FLT3 expression. It seems, therefore, that low MYB activity could be sufficient to maintain FLT3 expression in these cells. Likewise, the down-regulation of FLT3 expression in the *Myb*-deleted LMPP fraction of the bone marrow seemingly contrasts with the fact that the *Myb* ^Plt4/Plt4^ mutant mice present normal levels of FLT3 expression. A notable particularity of the latter model is that the animals retain a small number of CLP [32], which are typically lost upon *Myb* deletion. It thus constitutes an attractive model to study MYB involvement in gene regulation associated with the lymphoid priming of multi-potential progenitors and was used to pinpoint MYB-dependent *Il7r*α up-regulation as a crucial event [31,32]. However, the ectopic expression of *Il7r*α failed to rescue lymphoid differentiation, suggesting that MYB may act at several levels to influence lineage commitment at this stage. The reason why *Myb* ^*Plt4/Plt4*^ LMPP display normal levels of FLT3 receptors may lay in the fact that MYB^PLT4^ mutant protein retains some ability to regulate *Flt3* expression. Alternatively, the combined contribution of other regulators, including HOXA9, MEIS1, PBX1 C/EBPα and PU.1, may adjust over time to compensate for the effect of perturbed MYB^PLT4^ activity on *Flt3* regulation. These key factors have a determining role for HSC maintenance and function. Others have previously demonstrated the role of HOXA9 in lympho-haematopoietic progenitor function and *Flt3* regulation [38]. PU.1 function was shown to be crucial during the transition from the HSC to the earliest myeloid and lymphoid progenitors [39], whilst later stages of B-cell maturation can progress in its absence. C/EBPα expression in multipotent HSC was identified as a component of a flexible mechanism for lineage priming and fate commitment [40] and was shown to participate in regulating HSC proliferative capacity [41,42]. We previously found that both MYB and C/EBPα could independently activate *FLT3* expression in AML [17]. Here, we show that while single knock downs of either factor have no effect on FLT3 expression in primary KSL cells, co-silencing of *Myb* and *Cebpa* expression leads to a reduction in the proportion of the FLT3^+^ KSL cells. Using cell line models, we further highlight HSC-specific characteristics that include the need for the functional cooperation of both factors in order to fully activate *Flt3* transcription. Together with MYB, both C/EBPα and PU.1 were shown to cooperate in the regulation of myeloid-specific genes such as myeloperoxidase and neutrophil elastase [43,44]. However, to our knowledge, this is the first example of such synergy in the HSC compartment. We further demonstrate that the full realization of this cooperation involves the *Flt3* HS-C intronic element. The significance of this region for *Flt3* regulation is also highlighted at the transition from HSC to multipotent progenitors. Using primary HSC and progenitor cells, we uncovered a two-step mechanism underlying *Flt3* regulation at the early stages of normal haematopoiesis. Prior to detection of FLT3 expression at the cell surface, the accessibility of the promoter region appears to reflect a primed state of the *Flt3* gene in the most immature fraction of the HSC compartment. This open state of the *Flt3* promoter, which is also seen in CMP and GMP, may allow basal transcriptional activity and is consistent with previous studies reporting low levels of *Flt3* RNA in KSL/FLT3^-^ multipotent HSC and early GMP [45,46]. The accessibility to the intronic site characterises the cells actively expressing the receptor.

While the importance of MYB for LMPP function as been previously established, the mechanisms through which MYB influences lineage commitment at this stage are not clear. In fact, both disruption the *Myb* gene and enforced expression of the protein in LMPP skews differentiation towards myeloid rather than lymphoid cells [31]. We find that, together with an effect on *IL7r*α, *Flt3* up-regulation is another determinant of MYB-dependent molecular events required for lymphoid priming of the MPP. We also find that, *Flt3* transcriptional regulation in uncommitted cells relies on a functional cooperation between MYB and C/EBPα.

## Supporting Information

S1 Fig Induced *Myb* deletion does not lead to increase apoptosis in KSL/progenitor cells. Representative histogram and two-dimensional flow cytometric plot analysis of cells in the lineage negative, progenitors and KSL compartment of the bone marrow of the cKO (*Myb* ^*F/F*^:*MxCre*) and control (*Myb* ^*+/+*^:*MxCre*) mice 24 (A) and 48 (B) hours post poly(I:C) injection. Histograms represent the percentage of apoptotic cells within lineage negative, progenitor and KSL populations, with numbers presented as mean ± SEM, determined from 3 independent experiments.
(TIF)

S2 Fig Analysis of the KLS compartment 24 hours post MYB deletion using the CD150/CD48 strategy. Representative two-dimensional flow cytometric analysis of cells in the KSL compartment of the bone marrow of the cKO (*Myb* ^*F/F*^:*MxCre*) and control (*Myb* ^*+/+*^:*MxCre*) mice 24 hours post p(I:C) injection. The proportion of LMPP-enriched KSL was assessed based on the surface expression of CD48 and CD150. Serial gating defined a population highly enriched in LMPP (CD150^+/-^CD48^+^KSL) for which the percentage of Flt3^hi^ cells was measured. Regrouping 3 independent experiments, the right panel shows the percentage of FLT3 expression in the gated population, indicated as plot header. Numbers are plotted as mean ± SEM (***, p<0.0001).
(TIF)

S3 Fig Luciferase reporter studies in non-haematopoietic 293T cells. 293T cells were transfected using the TransIT-293 kit, according to the manufacturer’s instructions (Mirus Bio) with vectors encoding for MYB and C/EBPα proteins, together with the reporter constructs shown in Fig 5. Results were standardised to 100 and bars represent the average proportions across 3 experiments. All results are representative of 3 independent experiments.
(TIF)

S4 Fig Percentage of FLT3^+^ KSL in *Myb* haploinsufficient mice and wild type counterparts. Representative two-dimensional flow cytometry dot plots (upper panel) and box plot showing depicting the variation in FLT3^+^ cells within the KSL compartment of wild type and MYB+/- mice (lower panel).
(TIF)
