# Supplementary figures and images for: Regulation of the Flt3 Gene in Haematopoietic Stem and Early Progenitor Cells
Source: PLoS One. 2015 Sep 18;10(9):e0138257. doi: 10.1371/journal.pone.0138257 (PMC4575200; doi:10.1371/journal.pone.0138257)

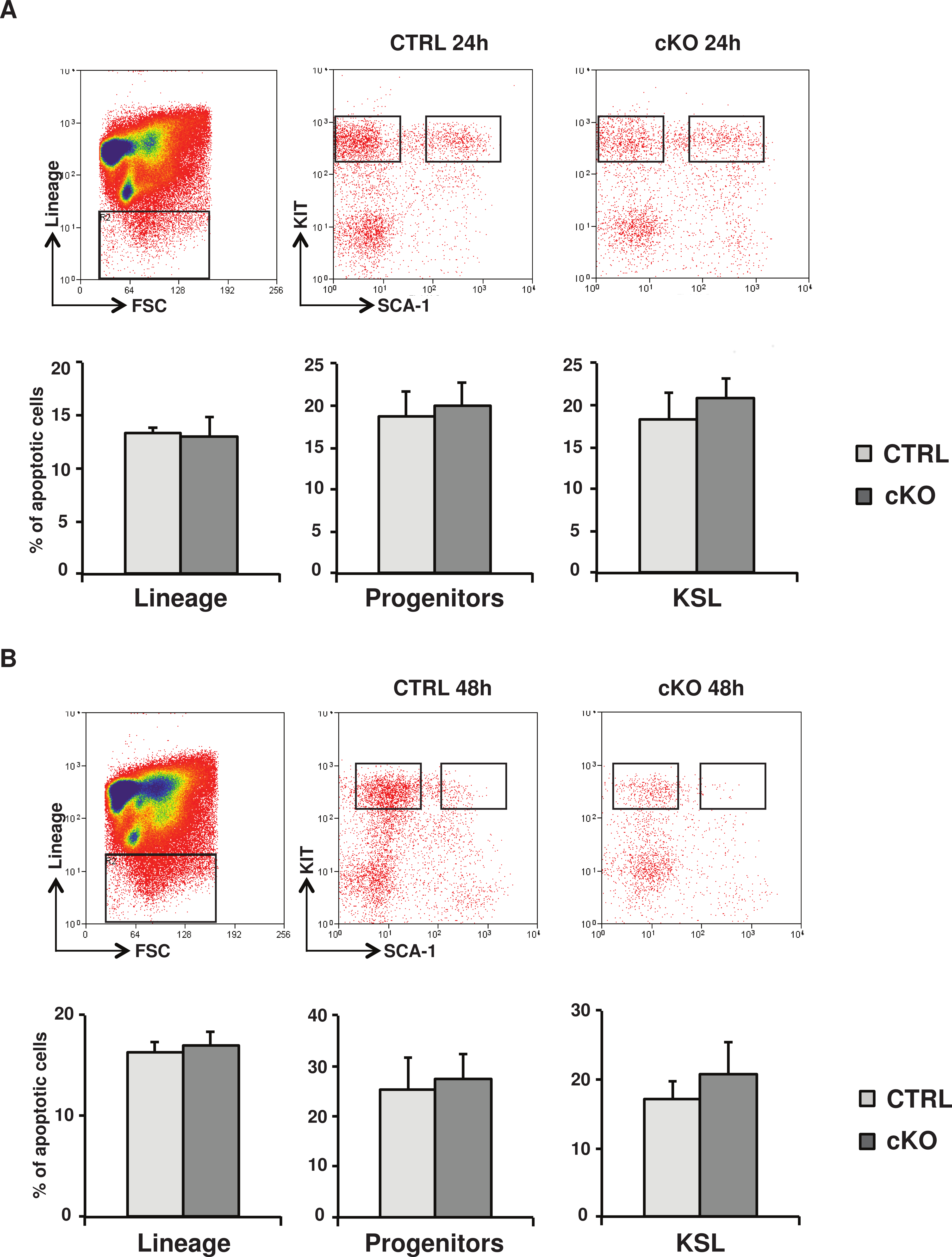

Supplement: S1 Fig — Representative histogram and two-dimensional flow cytometric plot analysis of cells in the lineage negative, progenitors and KSL compartment of the bone marrow of the cKO (Myb F/F:MxCre) and control (Myb +/+:MxCre) mice 24 (A) and 48 (B) hours post poly(I:C) injection. Histograms represent the percentage of apoptotic cells within lineage negative, progenitor and KSL populations, with numbers presented as mean ± SEM, determined from 3 independent experiments. (TIF) [file pone.0138257.s001.tif]

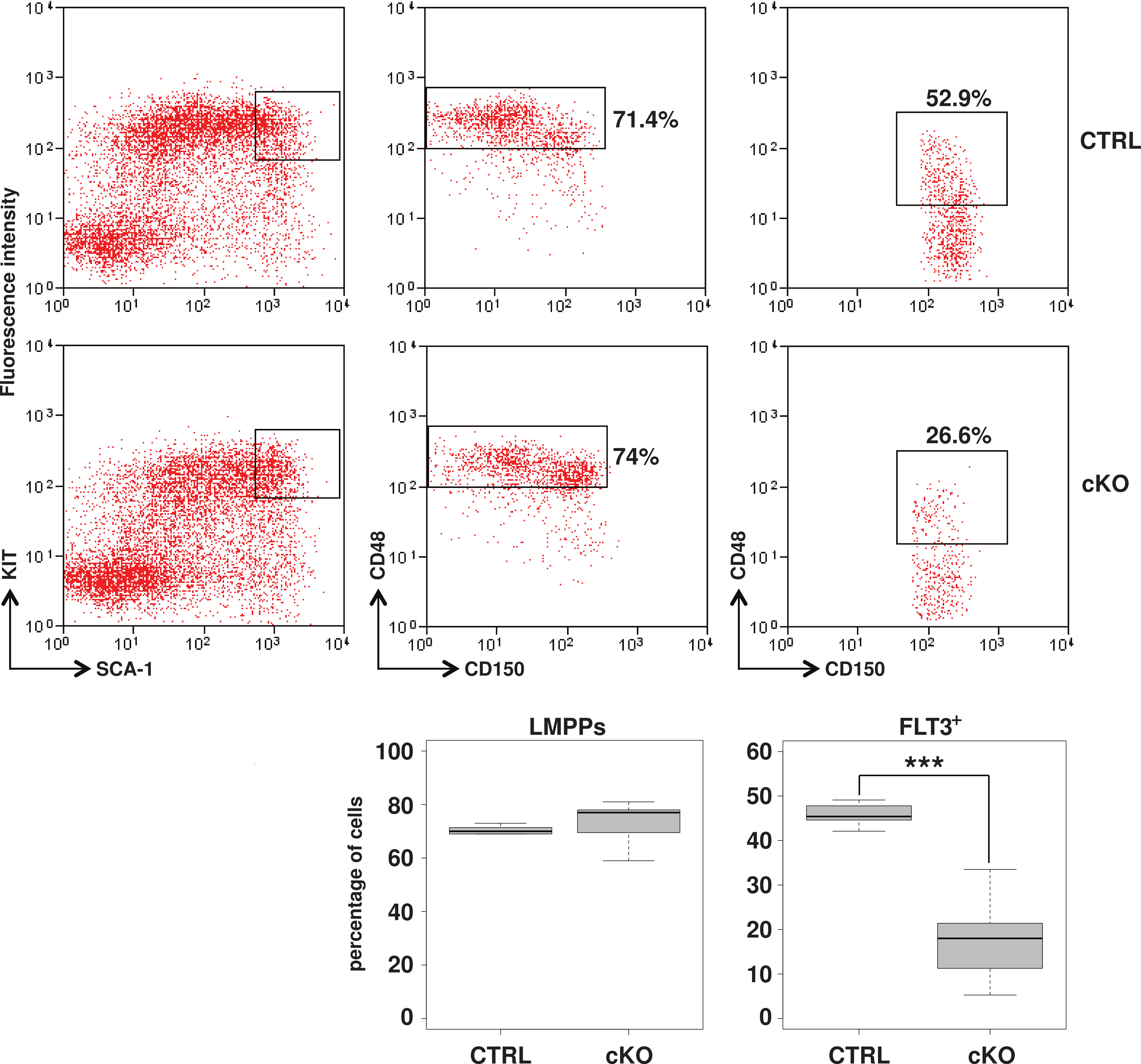

Supplement: S2 Fig — Representative two-dimensional flow cytometric analysis of cells in the KSL compartment of the bone marrow of the cKO (Myb F/F:MxCre) and control (Myb +/+:MxCre) mice 24 hours post p(I:C) injection. The proportion of LMPP-enriched KSL was assessed based on the surface expression of CD48 and CD150. Serial gating defined a population highly enriched in LMPP (CD150+/-CD48+KSL) for which the percentage of Flt3hi cells was measured. Regrouping 3 independent experiments, the right panel shows the percentage of FLT3 expression in the gated population, indicated as plot header. Numbers are plotted as mean ± SEM (***, p<0.0001). (TIF) [file pone.0138257.s002.tif]

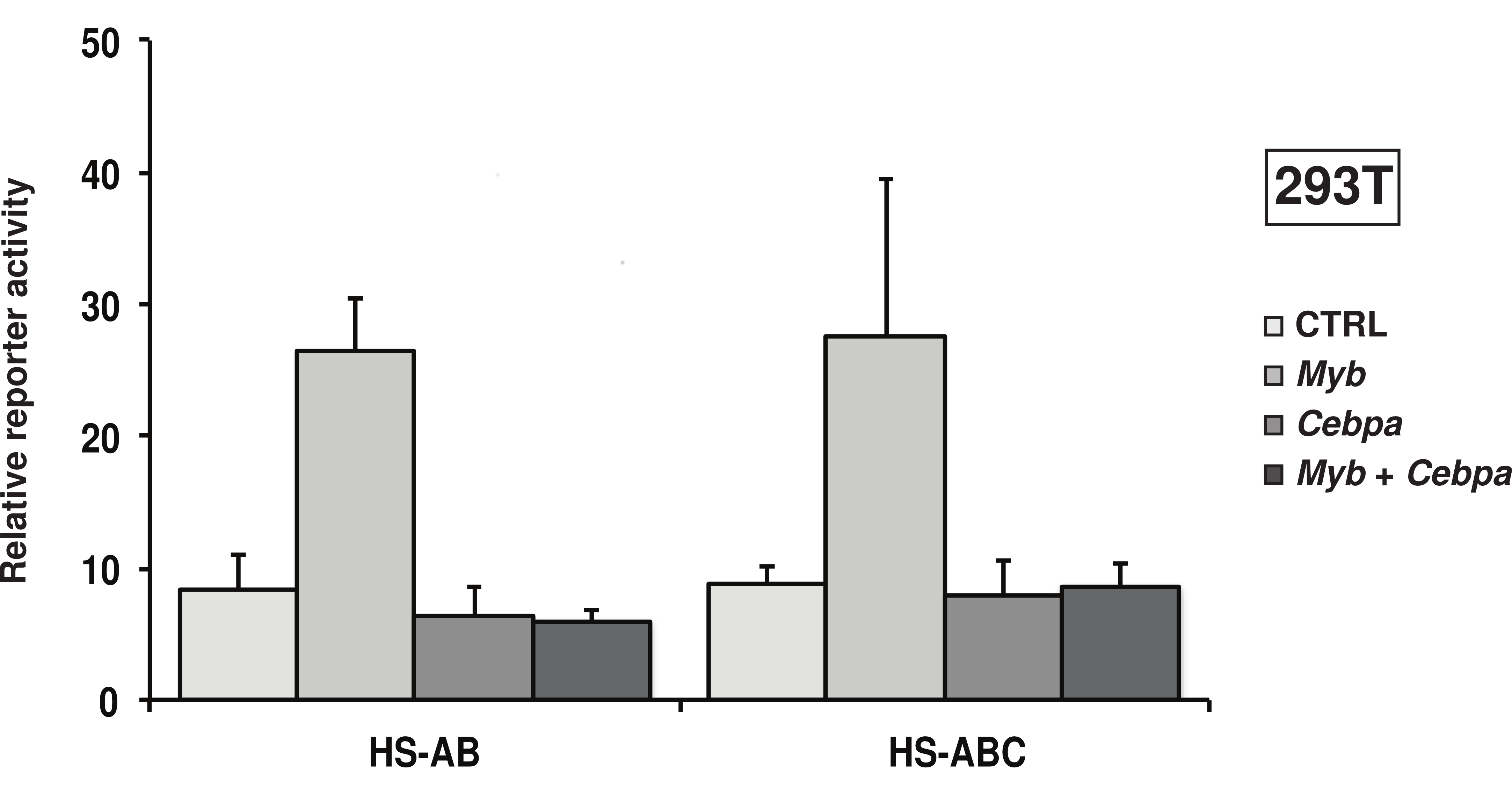

Supplement: S3 Fig — 293T cells were transfected using the TransIT-293 kit, according to the manufacturer’s instructions (Mirus Bio) with vectors encoding for MYB and C/EBPα proteins, together with the reporter constructs shown in Fig 5. Results were standardised to 100 and bars represent the average proportions across 3 experiments. All results are representative of 3 independent experiments. (TIF) [file pone.0138257.s003.tif]

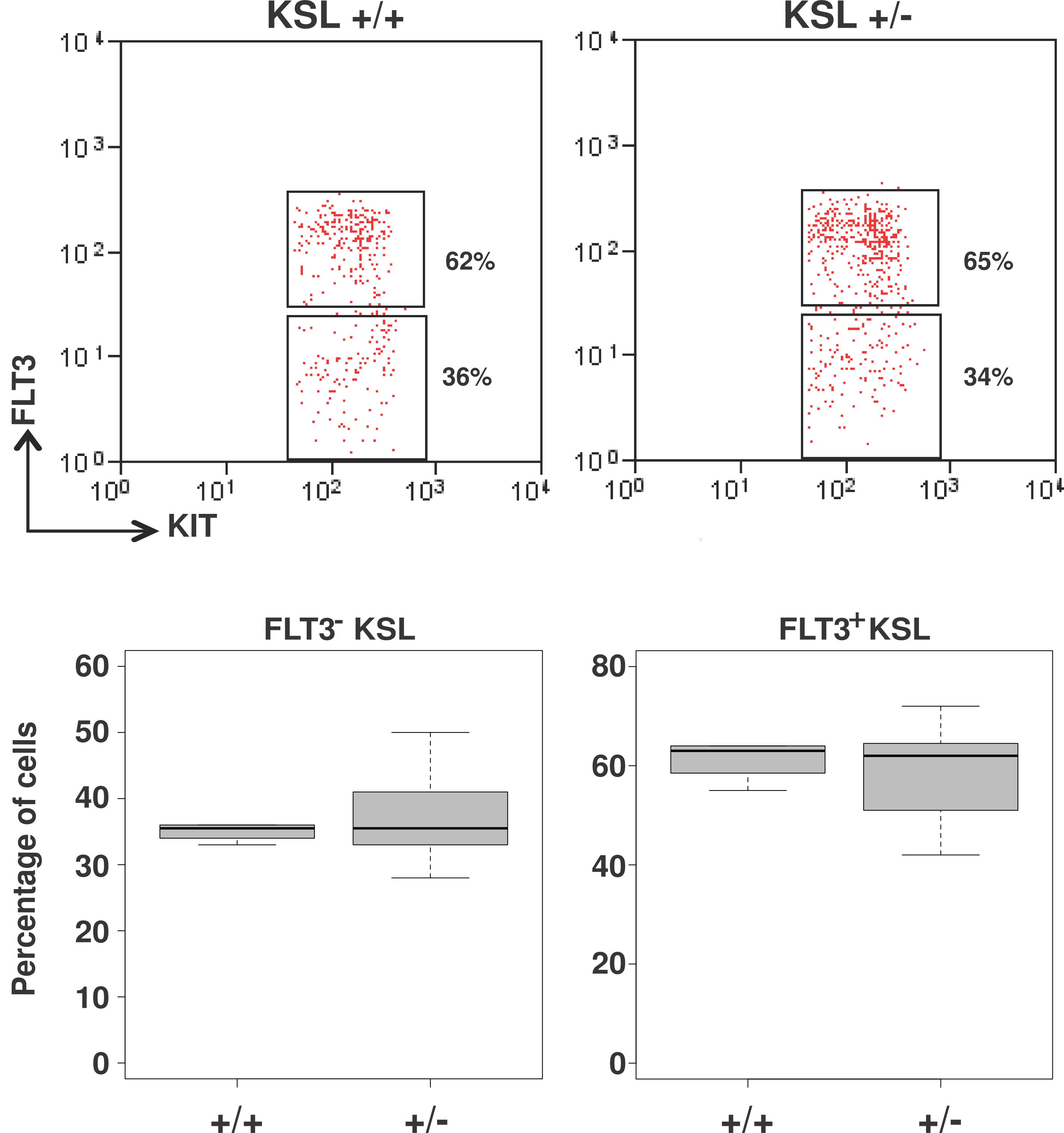

Supplement: S4 Fig — Representative two-dimensional flow cytometry dot plots (upper panel) and box plot showing depicting the variation in FLT3+ cells within the KSL compartment of wild type and MYB+/- mice (lower panel). (TIF) [file pone.0138257.s004.tif]
